# Development and Testing of Screen-Based and Psychometric Instruments for Assessing Resident Performance in an Operating Room Simulator

**DOI:** 10.1155/2016/9348478

**Published:** 2016-05-11

**Authors:** Richard R. McNeer, Roman Dudaryk, Nicholas B. Nedeff, Christopher L. Bennett

**Affiliations:** ^1^Department of Anesthesiology, University of Miami, Ryder Trauma Center, 1800 NW 10 Avenue, Miami, FL 33136, USA; ^2^Department of Biomedical Engineering, University of Miami, Ryder Trauma Center, 1800 NW 10 Avenue, Miami, FL 33136, USA; ^3^Music Engineering Technology, University of Miami, Frost School of Music, 1550 Brescia Avenue, Founder's Hall Rm 140, Coral Gables, FL 33146, USA

## Abstract

*Introduction*. Medical simulators are used for assessing clinical skills and increasingly for testing hypotheses. We developed and tested an approach for assessing performance in anesthesia residents using screen-based simulation that ensures expert raters remain blinded to subject identity and experimental condition.* Methods*. Twenty anesthesia residents managed emergencies in an operating room simulator by logging actions through a custom graphical user interface. Two expert raters rated performance based on these entries using custom Global Rating Scale (GRS) and Crisis Management Checklist (CMC) instruments. Interrater reliability was measured by calculating intraclass correlation coefficients (ICC), and internal consistency of the instruments was assessed with Cronbach's alpha. Agreement between GRS and CMC was measured using Spearman rank correlation (SRC).* Results*. Interrater agreement (GRS: ICC = 0.825, CMC: ICC = 0.878) and internal consistency (GRS: alpha = 0.838, CMC: alpha = 0.886) were good for both instruments. Subscale analysis indicated that several instrument items can be discarded. GRS and CMC scores were highly correlated (SRC = 0.948).* Conclusions*. In this pilot study, we demonstrated that screen-based simulation can allow blinded assessment of performance. GRS and CMC instruments demonstrated good rater agreement and internal consistency. We plan to further test construct validity of our instruments by measuring performance in our simulator as a function of training level.

## 1. Introduction

Medical simulation has become established as a safe and effective tool for training and assessing performance and competency in individuals and teams responsible for patient care [1–4]. It is not uncommon for clinicians-in-training to first practice difficult airway management skills [4] and life support algorithms [5] on simulated patients before performing in actual clinical settings on patients. Increasingly, simulation-based studies are being used to test hypotheses [6–11]. For example, two recent reports studied the impact of stress training on surgical and anesthesia resident performance by experimentally controlling the stress content of simulated emergency scenarios [6, 7]. Simulation has an important role to play in hypothesis-driven experimental design, because hypotheses can be tested without exposing patients and workers to risk, and simulated conditions are more controllable than the complex and unpredictable conditions inherent in the clinical arena.

A fundamental requirement common to the interpretation of simulation outcomes is that the assessment instruments selected be validated. A newly conceived instrument, such as one to assess clinical performance, may have latent flaws that must be identified and corrected before implementation [12]. Instrument items in the form of survey questions, for example, may be prone to inconsistent or ambiguous interpretation by raters. Generally, instruments for assessing competency are first conceived based on expert opinion and validated for construct validity, internal consistency, and interrater reliability using an iterative process involving sequential experiments [13]. Only after an instrument has been validated should it be used in formal experiments. Although simulation-based experiments can be designed to be prospective, randomized, and double-blinded, thus, abiding by principles of medical research, performance in simulators is often assessed by direct observation of subjects or viewing of recorded video of subjects [14, 15], making it difficult to blind researchers and/or expert raters to experimental condition and subject identity.

We describe the development of methodology for assessing subject performance using a screen-based simulator in the setting of a simulated operating room. We designed our screen-based interface to allow subjects to log their observations, proposed interventions, and courses of action during simulated intraoperative emergencies. An important benefit of this interface is that expert raters are able to later review the responses logged by subjects while remaining completely blinded to subject identity, gender, and experimental condition.

Screen-based simulation has had an important role in the field of anesthesiology [16] including recent reports that address its impact in education [17] and in patient care [18]. The methodology we report here promotes a role for screen-based simulation in the conduct of randomized controlled experiments in which blinded assessment of subjects is required. Therefore, the primary objective of this paper is to describe our methodology and “test-drive” it in a preliminary experimental setting. In designing the experiment, we developed a Global Rating Scale and Crisis Management Checklist, and though these were adapted from previously validated instruments [19], they need to be refined and validated before being considered in formal experiments. Therefore, our secondary objective is to report the results of the first iteration in the validation of these instruments, which will be useful in future validation experiments and supply effect size and variance parameters needed to calculate sample size in the design of formal experiments.

## 2. Methods

### 2.1. Experimental Design and Study Population

The institutional review boards at the University of Miami and Jackson Health system reviewed and approved this study. Twenty first-year clinical anesthesia (CA-1) residents participated in this study after informed consent.

### 2.2. Graphical User Interface

**Figure 1:**
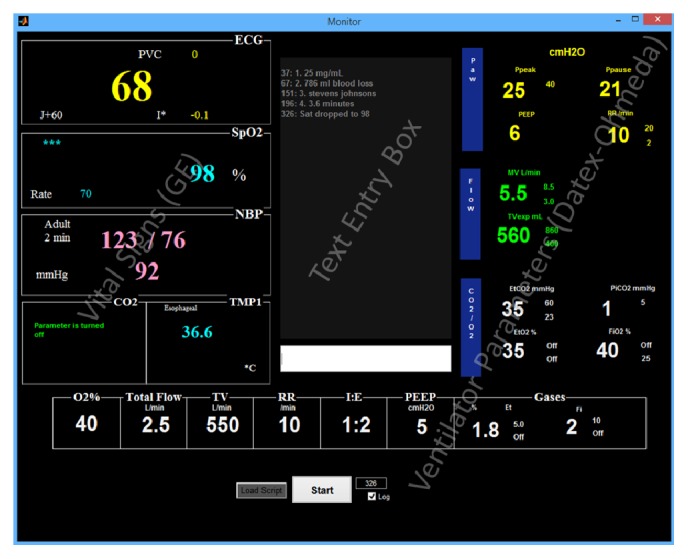
Graphical user interface (GUI) used in simulation experiments. Parameters were updated at one-second intervals based on values read from an XLS file. The GUI featured a responsive pulse oximeter auditory display and IEC alarms that would annunciate when parameter alarm thresholds were transcended. Subjects entered answers to distractor questions and responses to state changes in the text entry box.

A custom graphical user interface (GUI) was developed in the MATLAB (MathWorks, Natick, MA) environment. The GUI frontend was designed to combine the displays of the GE monitor and Datex-Ohmeda ventilator into a single display (Figure 1). The GUI was programmed to read simulation scripts stored in XLS file format and update displayed GUI parameter values to reflect scripted values. When reading an XLS script, the GUI played script-dependent pulse oximeter pulse tone and audible alarm annunciation through a PC speaker. Alarm annunciation depended on scripted values reaching predefined threshold values within the XLS script (see Section 2.3). Additionally, the GUI had a text entry box allowing text responses to be entered by subjects (Figure 1), and these responses were logged with timestamps into a separate file (CSV file format) which was used after completion of experiments to construct stem plots used by raters (see Section 2.5).

### 2.3. Simulation Scripts

**Figure 2:**
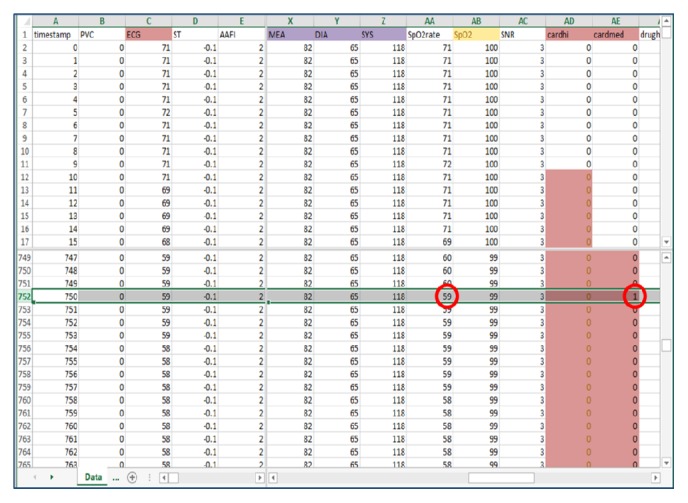
Screenshot of a portion of the “symptomatic bradycardia” XLS script. Note cell AA752 which shows the first time the heart rate drops below 60 bpm and surpasses an alarm threshold. Cell AE752 programmatically changes to a value of 1 which instructs the GUI to annunciate the appropriate IEC alarm, in this case the medium priority cardiac alarm “cardmed.”

**Figure 3:**
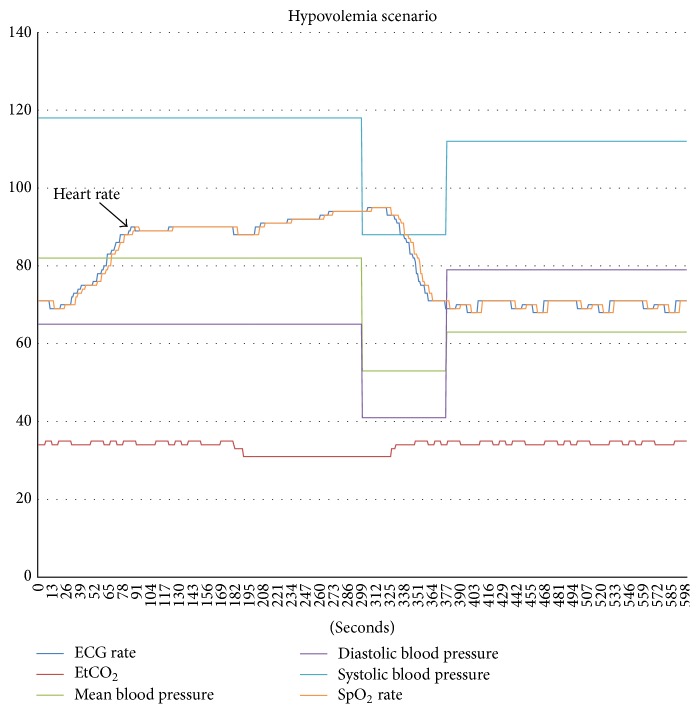
Plot showing the changes to relevant parameters in the “hypovolemia” scenario. Near the beginning, heart rate gradually increases over 5 minutes but does not surpass the alarm threshold. Later in the scenario, a low blood pressure is measured and the appropriate alarm sound enunciated. All parameters normalize and revert back to baseline levels before the end of the scenario.

Simulation scripts were conceived and written in XLS format (Figure 2). The file layout consisted of a timestamp column (with 1-second intervals) and subsequent columns for each of the simulated parameters. Additional columns were used to represent the annunciation of the standard International Electrotechnical Commission (IEC) 60601-1-8 alarms [20] based on commonly used adult-patient alarm thresholds. Cells under the alarm columns had default values of 0 indicating an alarm-off state, and when an alarm threshold was reached, the corresponding alarm cell value would programmatically change to 1, and the GUI would annunciate the corresponding IEC alarm. Each script simulated a 30-minute lunch break and had a total of 1800 rows (seconds). Two “uneventful” scripts consisted of normal vital signs and ventilator parameters. Two “eventful” scripts contained three intraoperative events each. The first eventful lunch break simulated (i) circuit disconnect in the first ten minutes, (ii) symptomatic bradycardia in the second ten minutes, and (iii) endobronchial tube migration in the last ten minutes. The second eventful lunch break simulated (iv) hypovolemia in the first ten minutes, (v) pulmonary embolism in the second ten minutes, and (vi) light anesthesia in the third ten minutes (see Figure 3 for “hypovolemia” scenario). In between each intraoperative event vital signs and ventilator parameters returned to normal.

### 2.4. Distractor Task Questions

A set was created of 100 questions relating to the practice of anesthesiology. The questions were menial and tedious, usually requiring simple calculations to be performed in order to arrive at the answer. For example, “calculate the BMI for a 28 yo female who is 5 foot 9 inches and 225 pounds,” “calculate the paO2/FiO2 ratio when paO2 = 107 mm Hg and FiO2 = 80%,” and “during general anesthesia, a mixture of 60% N2O and 40% O2 is being administered to a patient. Assuming the flow rate of O2 is 2 liter/min, what is the flow rate of N2O?” Some questions required reference to a pharmacopeia, for example, “what is the renal dosing for tiagabine? You can use computer/phone (e.g., Epocrates*™*).”

### 2.5. Performance Assessment

**Figure 4:**
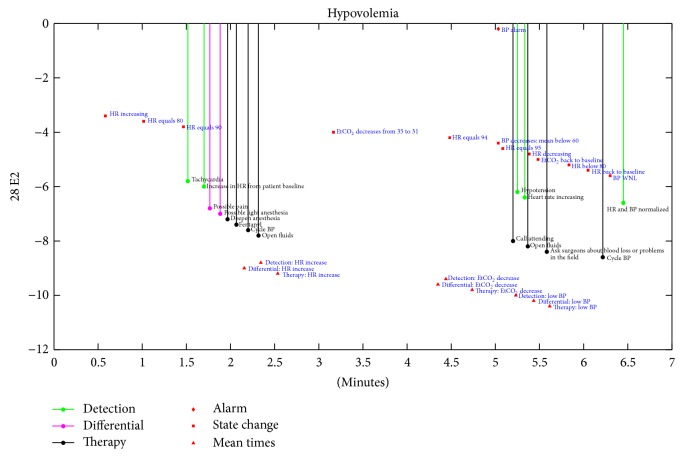
Stem plot showing the responses entered by a subject into the GUI during the “hypovolemia” scenario. Note that the *y*-axis scale does not have any informative value. The diamond and square red markers represent the times when state changes and alarm annunciation occur in the script. The filled circles are color coded based on the legend and show the relative times and text responses entered by subjects. The red triangles represent the average times subjects took to detect, diagnose, and treat scripted problems.

In order to allow expert raters to review subject responses during emergencies in a blinded fashion, stem plots were generated displaying subject responses into three categories of information (Figure 4). The first category was the timing of when scripted state changes (e.g., “HR increasing”) and alarm annunciation (e.g., “BP alarm”). This group of information was located near the top of each stem plot, and the timing and occurrence of these events were constant for all subjects. The second group of information contained individual subject text entries and was located midway between the top and bottom of the stem plots, allowing raters to easily review the extent, order, and timing of subject responses. The third group of information showed the average times subjects took to respond with detection of state change(s), differential, and intervention(s) and was located near the bottom of the stem plots. The three groups of information were thus distinguishable by area location on stem plots and, additionally, stem color and symbol (see Figure 4).

The Ottawa Crisis Resource Management Global Rating Scale and Simulation Session Crisis Management Skills Checklist [19] were adapted for this study. A three-member team of board-certified anesthesiologists including a member responsible for running our department's simulation-based curriculum adapted the Global Rating Scale and Crisis Management Checklist. The Global Rating Scale (see Appendix) contains five items on a seven-point Likert scale. Each item contains an item-specific description of how to choose the Likert intervals. One item rated overall performance and three rated state change (e.g., alarm) detection, situational awareness, and resource utilization. A fifth item was included to rate subject perception of the extent to which an emergency had been resolved.

The Crisis Management Checklist consists of three subscales for the assessment of the ability to detect state changes, to be situationally aware, and to initiate proper therapy or interventions. Each of these categories has individual items related to timeliness, completeness, appropriateness, and prioritization. Raters can score each item trichotomously as “yes” (2 points), “marginal” (1 point), or “no” (0 points). Some items such as “missed detection” are scored with negative points.

Using the Global Rating Scale and Crisis Management Checklist assessment instruments, subject performance was evaluated by two raters with clinical expertise in anesthesiology who were blinded to the experimental condition and subject identity. The reviewers were asked to examine the stem plots of the subject responses logged during simulation experiments (see Figure 4 for an example pertaining to the “hypovolemia” scenario). Calibration of raters was accomplished by having the two raters as a group assess performance for four subjects who participated in a preliminary stage of this project and who did not participate in the current study. After calibration, raters independently assessed performance for the 20 enrolled subjects.

### 2.6. Statistical Analysis

All statistical analyses were performed using SPSS software suite (IBM®). Interrater reliability was assessed by calculating the intraclass correlation coefficient (ICC) [21] between responses of expert raters on both the Global Rating Scale and Crisis Management Checklist instruments (two-way mixed, absolute agreement). The ICC was calculated as an aggregate for all scenarios and for each scenario separately. Internal consistency of the Global Rating Scale and Crisis Management Checklist instruments was assessed by calculating the corrected item-total correlation and Cronbach's *α* [22] from the average of expert rater responses. Internal consistency was determined based on an aggregate of all scenarios and for each scenario separately. Spearman rank correlation was calculated to assess agreement between the Global Rating Scale and Crisis Management Checklist from the average of the rater responses.

## 3. Results

### 3.1. Internal Consistency and Interrater Reliability

The two expert raters assessed subject performance using the Global Rating Scale and Crisis Management Checklist. Each subject was rated 6 times per rater, once for each scenario, and the total number of ratings from each rater on 20 subjects was 120. Tables 1 and 2 show the degree to which the Global Rating Scale and Crisis Management Checklist measure the same construct, respectively, based on average ratings by both raters. Internal consistency was “good” (Table 1) for the Global Rating Scale (*α* = 0.838) and items showed good discrimination except for the “subject perception of crisis resolution” item with a corrected item-total correlation of 0.117. When this item is removed, Cronbach's *α* increases to 0.930 (“excellent”). Cronbach's *α* also indicated “good” internal consistency (*α* = 0.886) for the Crisis Management Checklist (Table 2). Two items possessed Item discrimination values less than 0.3 (“one or more incorrect diagnoses” and “one or more inappropriate actions”). Removal of these items results in modest increases in Cronbach's *α*.

**Figure 5:**
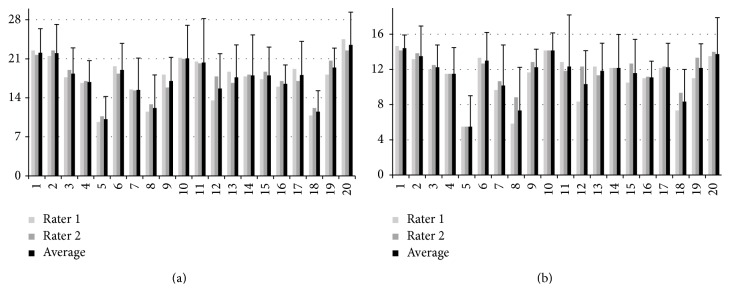
Overall subject performance assessment scores from the Global Rating Scale (a) and Crisis Management Checklist (b). Individual rater and average ratings are shown. The bars depict standard deviations.

Tables 3 and 4 summarize interrater agreement for the Global Rating Scale and Crisis Management Checklist, respectively, as assessed by intraclass correlation. Good agreement was observed between raters for the Global Rating Scale when all emergency scenarios were considered collectively (see last column in Table 3 and Figure 5(a)). The lowest agreement was observed for the “subject perception of crisis resolution” item (ICC = 0.624). Considering each emergency scenario separately, total agreement was the highest for the “pulmonary embolism” scenario (ICC = 0.899) and the lowest for the “light anesthesia” scenario (ICC = 0.760). For three of the emergency scenarios (“hypovolemia,” “pulmonary embolism,” and “light anesthesia”) the ICC was insignificant (*P* > 0.05) for the “subject perception of crisis resolution” item. Modest increases in total ICC are observed when this item is removed from consideration (bottom row in Table 3).

Interrater agreement when considering all scenarios was good for each subscale in the Crisis Management Checklist (Table 4 and Figure 5(b)). The lowest item agreement in the “state change detection” scale was observed for the “missed detection” item, in the “situational awareness” scale it was the “one or more incorrect diagnoses” item, and in the “therapy/resource management” scale, it was the “one or more inappropriate actions” item. Each of these items also had low, insignificant, or incalculable ICC when emergency scenarios were considered separately. When these items are removed, ICC generally increases modestly for subscales and the total ICC.

### 3.2. Construct Validity

**Figure 6:**
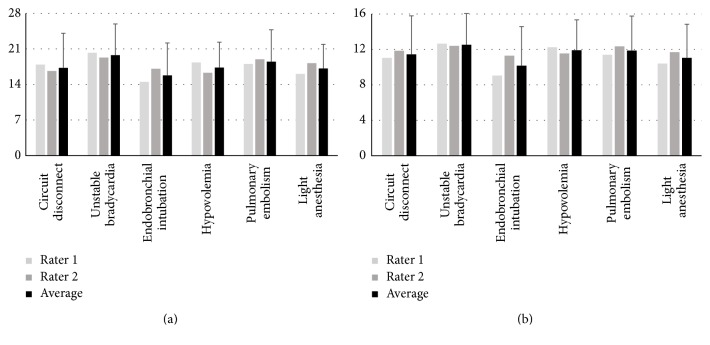
Subject performance assessment scores from the Global Rating Scale (a) and Crisis Management Checklist (b) based on emergency scenario. Individual rater and average ratings are shown. The bars depict standard deviations.

Correlation between the Global Rating Scale and Crisis Management Checklist total scores (averaged across all six emergency scenarios) was high (Spearman rank correlation = 0.948, *P* < 0.0001, *N* = 20 subjects) indicating good convergent validity (see Figure 5). Additionally, good agreement between the Global Rating Scale and Crisis Management Checklist total scores based on emergency scenario grouping averaged across subjects (Figure 6) was observed (Spearman rank correlation = 0.943, *P* = 0.005, *N* = 6 emergency scenarios). Considerable intersubject variability was observed with total scores ranging from 10.2 (4.1) to 23.5 (5.9) for the Global Rating Scale and from 5.5 (3.5) to 14.4 (1.5) for the the Crisis Management Checklist.

*Estimation of Effect Sizes*. Estimated effect size and variability were calculated in three arbitrary ways. First, aggregate subject scores were divided into two groups—the lower and upper 50th percentile based on the median—and averaged. Second, aggregate scores were divided into the 2nd and 3rd quartile. And finally, scores obtained during the “symptomatic bradycardia” and “endobronchial intubation” scenarios were treated as two groups and averaged.  Table 5 shows these mean scores, standard deviations, raw differences, percent differences, and Cohen's *d* values.

## 4. Discussion

Simulation-based experiments offer a viable controlled strategy to test hypotheses and interventions before implementation in actual clinical settings. We are interested in developing and testing techniques for characterizing the impact of intraoperative factors on anesthesiologist performance and patient safety. We have developed a novel screen-based interface and adapted previously validated Global Rating Scale and Crisis Management Checklist instruments for assessing performance in our simulator. Based on the results presented here, the feasibility of this methodology as a tool for allowing blinded assessment of subjects by expert raters has been demonstrated. Additionally, the first step in validating our performance assessment instruments has been accomplished.

One of the fundamental features of our screen-based interface is that expert raters assess performance based on subject responses and actions logged through the interface, assuring that raters are blinded to subject identity and experimental condition. Automated timestamping of the logged responses facilitated the assembly of the stem plot timelines (see Figure 4) which were crucial to expert raters when completing the Global Rating Scale and Crisis Management Checklist instruments. It is likely that these methodology features and the fact that raters were trained in instrument use prior to beginning this pilot study contributed to the good interrater reliability observed for both the Global Rating Scale and Crisis Management Checklist instruments.

Internal consistency was good for both the Global Rating Scale and Crisis Management Checklist instruments, as assessed with Cronbach's *α* (*α* = 0.838 and 0.886, resp.), supporting their reliability; for comparison, Gerard et al. (2013) measured a Cronbach's *α* of 0.77 for a checklist that assesses lumbar puncture proficiency in pediatric residents [13]. However, several instrument items should be considered for removal from our instruments because of low item discrimination values (in the Global Rating Scale: “subject perception of crisis resolution” and in the Crisis Management Checklist: “one or more incorrect diagnoses” and “one or more inappropriate actions”). Additionally, these items and the “missed detection” item in the Global Rating Scale demonstrate inconsistent interrater agreement when emergency scenarios are considered separately. Removal of these items leads to modest increases in internal consistency and interrater reliability. These items likely do not align with the other items in measuring the same construct (presumed to be performance) and/or were ambiguously interpreted by raters, and they will be removed in the next iteration of our instruments.

The high correlation (0.948) between the Global Rating Scale and Crisis Management Checklist ratings suggests good convergent validity for the two instruments. However, it has been pointed out that caution in interpreting this result is warranted because the same expert raters assessed subject performance with both instruments, and, as a result, scores for each instrument cannot be assumed to be truly independent of the other [13]. Discriminant ability, which is another indication of construct validity, is harder to appreciate due to the fact that our experimental setup was not appropriately designed and powered for making this assessment. The ability to detect differences in performance would have been enhanced if subjects could be reliably grouped based on level of training and clinical experience; our subjects were all CA-1 anesthesia residents. However, discriminant ability is supported (albeit modestly) by the observations of a wide range of scores across the 20 subjects, the high interrater agreement for both the Global Rating Scale and Crisis Management Checklist, and the good agreement (high correlation) between the Global Rating Scale and Crisis Management Checklist.

With revised assessment instruments, the next experiments will be guided in part by the effect sizes and variability of responses observed here. Though not optimal, we chose to roughly estimate (possible) effect sizes by comparing subject scores between two groups that straddle the median total score (averaging across all emergency scenarios) (Table 5). We plan to test the discriminant ability of the revised instruments on a new group of subjects of differing training level (e.g., CA1s versus CA3s). Those experiments will help to further refine our expectation of effect sizes.

Screen-based simulation is considered to be less realistic than mannequin-based simulation; however, the utilization of our interface, within the context of a fully functional replica of an OR which included a METI mannequin, likely helps mitigates this penalty. Mannequin-based simulators may be better at assessing behavioral outcomes dealing with leadership, group dynamics, and communication skills than screen-based simulators, but the outcomes for this pilot study dealt with individual performance in management of intraoperative emergencies, and evidence exists where screen-based simulation can be effective at allowing assessment of performance in crisis management training [11, 23].

In addition to limitations discussed above, a fundamental limitation of the current study stems from the fact that, at this pilot stage, there is no way to ascertain that the construct measured by the Global Rating Scale and Crisis Management Checklist actually equated to subject performance. Relative to simulation for education, there are numerous challenges inherent in using simulation as a tool for assessment [24]. In our preliminary experiment, subject “performance” may have been influenced by the variability of previous exposure of residents to our institution's simulation curriculum, for example. Additionally, resident typing and computer skills could impact rated performance in our screen-based experimental setup. Again, we are planning a study to test performance as a function of resident year as assessed by our instruments which will help clarify the linkage of performance to construct measurement by our instruments.

We have previously shown that intraoperative noise increases anesthesia resident perception of fatigue and task load in an OR simulator that approximates environmental conditions in our clinical ORs [25, 26]. Our long-term goal with respect to the development of the screen-based interface, Global Rating Scale, Crisis Management Checklist, and other related methodologies described in this paper will be to test hypotheses and study the impact of environmental factors inherent to the OR on clinician performance outcomes.

## 5. Conclusions

We demonstrate the feasibility of a screen-based simulation experiment for blinded assessment of resident performance while managing intraoperative emergencies. Our modified global assessment and checklist instruments show good internal consistency, interrater reliability, and convergent validity. The next phase of experiments will be to determine discriminant ability of our setup in residents at different levels of training.

## Figures and Tables

**Figure 7:**
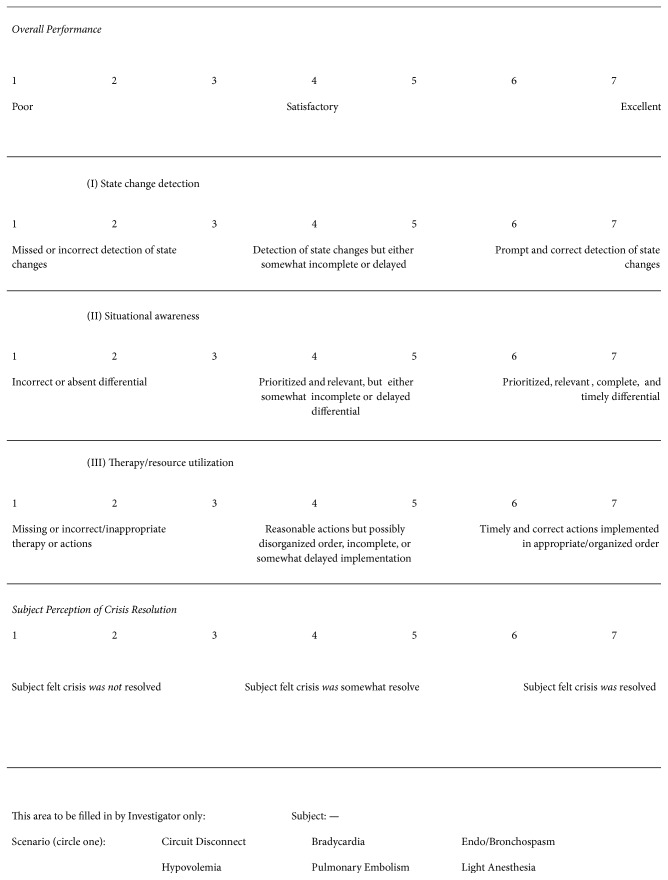
Global Rating Scale used by expert raters to assess performance of subjects in simulations.

**Figure 8:**
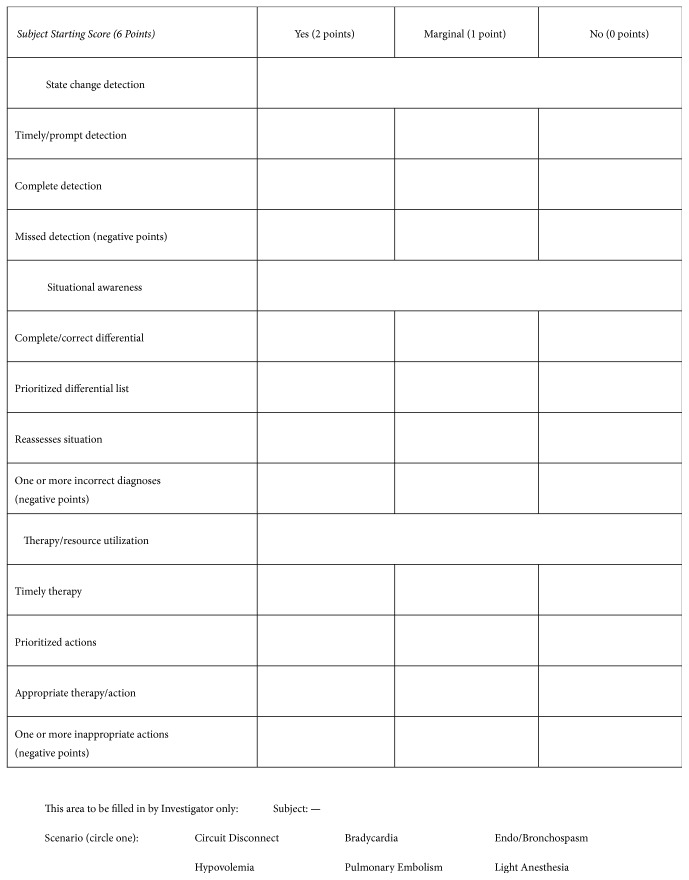
The Crisis Management Checklist used by expert raters to assess performance of subjects in simulations.

**Table 1:** Internal consistency of Global Rating Scale considering all emergency scenarios.

Item	Corrected item-total correlation^†^	Cronbach's *α* if item is deleted^#†^
Overall performance	0.909	0.726
State change detection	0.663	0.800
Situational awareness	0.828	0.747
Therapy/resource utilization	0.794	0.760
Subject perception of crisis resolution	0.117	0.930

^#^Cronbach's *α* = 0.838.

^†^Analysis performed on average of rater responses.

**Table 2:** Internal consistency of Crisis Management Checklist considering all emergency scenarios.

Item	Corrected item-total correlation^†^	Cronbach's *α* if item is deleted^#†^
State change detection		
Timely/prompt detection	0.457	0.885
Complete detection	0.545	0.879
Missed detection	0.533	0.881
Situational awareness		
Complete/correct differential	0.800	0.862
Prioritized differential list	0.774	0.864
Reassesses situation	0.759	0.867
One or more incorrect diagnoses	0.183	0.894
Therapy/resource utilization		
Timely therapy	0.696	0.870
Prioritized actions	0.771	0.864
Appropriate therapy/action	0.772	0.864
One or more inappropriate actions	0.177	0.893

^#^Cronbach's *α* = 0.886.

^†^Analysis performed on average of rater responses.

**Table 3:** Interrater agreement for the Global Rating Scale.

Item	Circuit disconnect	Bradycardia	Endobronchial intubation	Hypovolemia	Pulmonary embolism	Light anesthesia	All scenarios
Overall performance	0.756	0.900	0.772	0.789	0.908	0.844	0.804
State change detection	0.796	0.907	0.883	0.888	0.828	0.537^*∗*^	0.819
Situational awareness	0.889	0.848	0.872	0.673	0.964	0.835	0.866
Therapy/resource utilization	0.756	0.798	0.739	0.825	0.805	0.867	0.787
Subject perception of crisis resolution	0.683	0.633	0.740	0.529^*∗*^	0.414^*∗*^	0.538^*∗*^	0.624
Total^#^	0.798/0.852	0.899/0.906	0.812/0.860	0.849/0.838	0.892/0.929	0.760/0.826	0.825/0.856

Interrater agreement assessed by calculating the intraclass correlation coefficient (ICC) using a two-way mixed effects for consistency between two expert rater responses.

^*∗*^Not significant.

^#^The second ICC does not include the “subject perception of crisis resolution” item.

**Table 4:** Interrater agreement for the Crisis Management Checklist.

Item	Circuit disconnect	Bradycardia	Endobronchial intubation	Hypovolemia	Pulmonary embolism	Light anesthesia	All scenarios
State change detection^∧^	0.699/0.732	0.521^*∗*^/0.749	0.807/0.839	0.856/0.817	0.495^*∗*^/0.489	0.121^*∗*^/0.273^*∗*^	0.639/0.674
Timely/prompt detection	0.730	0.463^*∗*^	0.791	0.733	0.333^*∗*^	0.301^*∗*^	0.593
Complete detection	0.640	0.838	0.729	0.618	0.506^*∗*^	0.649	0.655
Missed detection	0.248^*∗*^	†	0.518^*∗*^	0.000^*∗*^	0.487^*∗*^	(−)0.366^*∗*^	0.088^*∗*^
Situational awareness^%^	0.835/0.838	0.946/0.952	0.834/0.856	0.773/0.773	0.907/0.937	0.798/0.803	0.844/0.852
Complete/correct differential	0.753	0.885	0.820	0.724	0.889	0.790	0.821
Prioritized differential list	0.710	0.913	0.790	0.825	0.857	0.794	0.807
Reassesses situation	0.733	0.387^*∗*^	0.739	0.533^*∗*^	0.647	0.708	0.620
One or more incorrect diagnoses	0.910	0.654	0.158^*∗*^	†	0.627	0.557	0.565
Therapy/resource utilization^&^	0.917/0.917	0.886/0.886	0.705/0.711	0.888/0.888	0.763/0.784	0.934/0.934	0.842/0.852
Timely therapy	0.945	0.647	0.594	0.795	0.798	0.871	0.793
Prioritized actions	0.857	0.681	0.570	0.914	0.733	0.840	0.780
Appropriate therapy/action	0.770	0.432^*∗*^	0.609	0.681	0.515^*∗*^	0.851	0.658
One or more inappropriate actions	†	†	0.000^*∗*^	†	0.487^*∗*^	†	0.314
Total^#^	0.903/0.898	0.954/0.965	0.871/0.860	0.917/0.931	0.870/0.908	0.850/0.881	0.878/0.890

Interrater agreement assessed by calculating the intraclass correlation coefficient (ICC) using a two-way mixed effects for consistency between two expert rater responses.

^*∗*^Not significant.

^†^Item responses were all zero.

^∧^The second ICC does not include the “missed detection” item.

^%^The second ICC does not include the “one or more incorrect diagnoses” item.

^&^The second ICC does not include the “one or more inappropriate actions” item.

^#^The second ICC does not include the previously excluded items.

**Table 5:** Estimation of effect sizes.

	Mean 1	Mean 2	Difference	% difference^∧^	Cohen's *d* ^!^
GRS					
Median^#^	15.1 (2.8)	20.2 (1.9)	5.1	25.2	1.5
Quartile^*∗*^	17.2	18.6	1.4	7.4	0.4
Scenario^&^	15.8	19.8	4.0	25.2	0.6
CMC					
Median^#^	10.0 (2.2)	13.0 (0.9)	3.0	23.3	1.3
Quartile^*∗*^	11.6	12.3	0.6	5.0	0.3
Scenario^&^	10.2	12.5	2.4	23.1	0.6

^#^Mean 1 and Mean 2 are average of scores from lower and upper 50th percentiles, respectively.

^*∗*^Mean 1 and Mean 2 are average of scores from 2nd and 3rd quartiles, respectively.

^&^Mean 1 and Mean 2 are average of scores from all subjects for “endobronchial intubation” and “unstable bradycardia” emergency scenarios, respectively.

^∧^Calculated as difference divided by average of means multiplied by 100.

^!^Calculated as difference divided by pooled standard deviation from all 20 subject scores.

## Appendix

See Figures 7 and 8.
